# Isoflurane post‐conditioning contributes to anti‐apoptotic effect after cerebral ischaemia in rats through the ERK5/MEF2D signaling pathway

**DOI:** 10.1111/jcmm.16282

**Published:** 2021-02-23

**Authors:** Qingtong Zhang, Jiangwen Yin, Feng Xu, Jingwen Zhai, Jieting Yin, Mingyue Ge, Wenyi Zhou, Nian Li, Xinlei Qin, Yan Li, Sheng Wang

**Affiliations:** ^1^ Department of Anesthesiology Lu’an Hospital Affiliated to Anhui Medical University Lu’an People’s Hospital Lu’an China; ^2^ Department of Anesthesiology First Affiliated Hospital School of Medicine Shihezi University Shihezi China; ^3^ Department of Anesthesiology First Affiliated Hospital of USTC Division of Life Sciences and Medicine University of Science and Technology of China Hefei China

**Keywords:** ERK5, ischaemia/reperfusion injury, isoflurane, MEF2D, neuroprotection

## Abstract

The mechanisms of brain protection during ischaemic reperfusion injury induced by isoflurane (ISO) post‐conditioning are unclear. Myocyte enhancement factor 2 (MEF2D) has been shown to promote neural survival in a variety of models, in which multiple survival and death signals converge on MEF2D and modulate its activity. Here, we investigated the effect of MEF2D on the neuroprotective effects of ISO post‐conditioning on rats after cerebral ischaemia/reperfusion (I/R) injury. Rats underwent middle cerebral artery occlusion (MCAO) surgery with ischaemia for 90 minutes and reperfusion for 24‐48 hours. After MCAO, neurological status was assessed at 12, 24 and 48 hours by the Modified Neurological Severity Score (mNSS) test. The passive avoidance test (PAT) was used to assess cognition function. Histological and neuropathological evaluations were performed with HE staining and Nissl's staining, respectively. We measured the expression of MEF2D, ERK5, GFAP and caspase‐3 by immunofluorescent staining and Western blotting, and TUNEL staining to assess the severity of apoptosis in hippocampal CA1 area. We found that MEF2D was involved in nerve protection after I/R injury, and post‐treatment of ISO significantly promoted the phosphorylation of ERK5, increased MEF2D transcriptional activity, inhibited the expression of caspase‐3 and played a role of brain protection.

## 1 INTRODUCTION

Ischaemic encephalopathy has the second highest fatality rate worldwide, according to a recent study.^1^ At present, the most efficient therapy for ischaemic encephalopathy is to restore blood supply as quickly as possible.^2^ However, blood recanalization may worsen brain damage and neurologic deficits. The phenomenon of aggravated function and structure of ischaemic brain tissue following blood reperfusion is called brain I/R injury.^3^ The mechanism of brain I/R injury involves numerous complex biological processes and signalling pathways,^4^, ^5^ including apoptosis.^6^, ^7^ Thus, it is important to illuminate the mechanisms of apoptosis and find efficient pharmacotherapies targeting the apoptotic process in brain I/R injury.

In the mammalian nervous system, the regulation of transcription factor activity is a key determinant of neuronal differentiation, survival and death.^8^ Activation of upstream signalling molecules to regulate transcription factors has become a particular research focus.^9^ MEF2s, originally identified as a nuclear factor in myogenic cells, have four different isoforms (MEF2A‐D).^10^ One of these family members, MEF2D, is mainly expressed in the cerebellum and hippocampus throughout the developing and adult brain.^11^ Numerous animal and human clinical experiments have suggested that the inhibition of MEF2D transcriptional activity can promote neuronal apoptosis. In the substantia nigra pars compacta (SNc) under stress conditions, MEF2D promotes dopaminergic (DA) neuronal survival.^12^ Although the role of MEF2D in neuronals has been studied, its role and regulatory mechanistic properties in the model of brain I/R injury are still unknown.

The concept of anaesthetic post‐conditioning is interesting in which its effects occur at critical moments at the start of brain I/R injury, and the activation of these neuroprotective mechanisms may outweigh the mechanisms that lead to I/R injury.^13^ Isoflurane (ISO) is an older inhaled anaesthetic drug frequently used in clinics.^14^ The mechanisms of its neuroprotective effect and ability to induce post‐conditioning are mainly related to the formation of reactive oxygen species, activation of cellular signalling pathways, and have an influence on mitochondria. Based on our previous studies, we showed that ISO post‐treatment plays a beneficial role in resisting brain I/R injury through the TGF‐β/Smad‐MAPK signalling pathway.^15^, ^16^

Despite the demonstrated protective effects of ISO, studies on ISO post‐conditioning activation signalling pathways are limited and deserve further study. One possible mechanism may be through the activation of extracellular signal‐regulated kinase 5 (ERK5). ERK5 is a crucial part of mitogen‐activated protein kinase (MAPK) family, which is regulated by a variety of mitogens and cell stress, and is participated in cell differentiation. Recent studies have shown that ERK5 is an important factor in cell survival.^3^ For example, Bickler et al have shown that ISO preconditioning increased intracellular p‐ERK5 expression by also increasing intracellular Ca2 + concentration temporarily, playing a crucial role in reducing neuronal death in oxygen‐glucose deprivation (OGD) models.^17^ The c‐terminal of ERK5 includes a transcriptional activation domain (aa 664‐789) and a MEF2 interaction region (aa 440‐501), which is critical for synergistic activation of MEF2.^18^ Studies have also shown that MEF2D is a specific substrate for ERK5^19^; additionally, expression and activation of MEF2D depends on the ERK5 mitogen‐activated protein (MAP) kinase.^19^ All of these provide the theoretical basis for the important role of ERK5/MEF2D signalling pathway in ISO post‐treatment of I/R injury.

In this study, we first sought to determine the neuroprotective effects of MEF2D in the model of I/R injury in rats. We further investigated the neuroprotective role of ISO in promoting transcriptional activity of its downstream transcription factor MEF2D by activating ERK5.

## 2 MATERIALS AND METHODS

### 2.1 Animals

Experiments were performed on 6‐ to 8‐week‐old male adult Sprague‐Dawley (SD) rats (220‐300g), as supplied by the laboratory animal centre of Shihezi University. Then, rats were divided into 9 groups randomly (n = 15‐20 each group): (1) Sham group, (2) I/R group, (3) shMEF2D + IR group , (4) shScr + IR group , (5)IR + ISO group, (6) shMEF2D + IR+ISO group, (7)shScr + IR+ISO group , (8) XMD8‐92 + IR+ISO group (X + IR+ISO) and (9) DMSO + IR+ISO group (D + IR+ISO). XMD8‐92 (5 μg/kg) was injected into the right ventricle at 30 min before MCAO (coordinate: A‐P −1mm, M‐L 1.5 mm, D‐V −4.5 mm).

We used a total of 187 rats. Rats with massive haemorrhage or complications of the middle cerebral artery during surgery were excluded. In all of the groups, the mortality rate was 6.4% (12 of 187), and the exclusion rate was 4.4% (7 of 158). The processing procedures of all animals were according to the National Institutes of Health (NIH) Guide for the Care and Use of Experimental Animals (80‐23, 1996). Study design and experimental methods were conformed to the Animal Ethics Committee of the First Affiliated Hospital of Shihezi Medical College.

### 2.2 In vivo MEF2D knockdown

To silence the expression of MEF2D, recombinant lentivirus vector expressing MEF2D‐shRNA was obtained commercially from Hanboi Biotechnology Co., Ltd., Shanghai, China. Lentiviral vectors coding for GFP were used as the control (Con‐shRNA). Twenty‐one days before MCAO, lentiviruses were injected into the ischaemic hippocampus as follows: A‐P −3.3 mm, M‐L 2.3 mm, D‐V −2.4 mm.

### 2.3 Model establishment

Middle cerebral artery occlusion (MCAO) was used to induce cerebral I/R injury, as described previously.^20^ Following anaesthetization by pentobarbital sodium (40 mg/kg) and buprenorphine‐HCl (0.1mg/kg) intraperitoneally, rats were kept on a thermostatic operating table at 37°C. After sterilizing the towel, the right neck skin was cut open to fully display the common carotid artery (CCA), internal carotid artery (ICA) and external carotid artery (ECA). Then, A 4‐0 line embolism (diameter with coating 0.37 ± 0.02mm; Doccol Corporation, Sharon, MA, USA) was sliped into the CCA and advanced ICA by 18.5 ± 0.5mm distance. After 90 min of ischaemia, the line embolism was removed for blood reperfusion. Rats assessed by Longa‐Z method without neurologic impairment after reperfusion (neurological function score < 1 or > 3) were excluded from this study.

### 2.4 Isoflurane post‐conditioning

Our primary intervention was ISO post‐conditioning. Following methods from our previous work,^21^ rats underwent 1.5% ISO post‐conditioning for 60 min, and the line embolism was removed at the beginning of reperfusion. Isoflurane concentrations were continuously monitored with an anaesthetic gas monitor (Drager Vamos, Germany).

### 2.5 Neurobehavioral assessment

After MCAO, neurological function was assessed 12, 24 and 48 h by the Modified Neurological Severity Score (mNSS).^22^ mNSS is a comprehensive test of sensation, reflection and balance. Neurological function is recorded from 0 to 18, where the higher the result, the more severe the injury. Sensorimotor deficit was determined by the adhesive‐removal test^23^: Two sheets of 113.1‐mm^2^ adhesive paper were attached to the distal radial area of the forelimb as bilateral tactile stimuli, and the time for each stimulus removal (maximum limit: 120 s) was recorded in three trials per day. Each test was at least 5‐min interval, and the rats were trained three days in advance of I/R injury.

### 2.6 Cognitive function detection

The passive avoidance test (PAT) is one of the most commonly used tools of cognitive function in experimental stroke studies.^24^ As rats show a tendency to prefer darkness, we designed an apparatus divided by a gate into two parts: a dark compartment and a brightly lit compartment. The floor of the dark compartment was made of electrified copper grid and connected to a stimulator. When rats entered the darkroom, an electrical shock (0.45 mA) was delivered to the grid in the dark compartment by the stimulator. Each rat was put in the brightly lit compartment, after 10 s, the gate was opened. The rat got the electric shock immediately when it entered the dark compartment. After 5 min, the rat was removed. The learning acquisition trial ended when the rat did not enter into the darkroom within 2 min. At 24 h following I/R injury, this procedure was repeated without the electric shock. We recorded the time taken (latency) for rats to enter into the darkroom and number of errors within 5 min. Experimenters were blind to the groups.

### 2.7 Cerebral infarction volume measurement

Twenty‐four hours after reperfusion, rats were decapitated and brains were quickly moved to the refrigerator at −20°C to freeze. After freezing, brain slices were prepared at 2 mm thickness and immersed in a 2% solution of TTC staining fluid (Sigma, St. Louis, MO, USA, T8877) at 37°C for 20 min. Red areas in the slices indicated normal brain tissue, and white area indicated ischaemic areas. The infarct volumes were analysed by ImageJ, and ischaemia area% = [(contralateral hemisphere area – non‐ischaemia area ipsilateral hemisphere area)/contralateral hemisphere area] ×100%.

### 2.8 Histological examination

After 24 h of reperfusion, the brain was severed after being fully anaesthetized with pentobarbital sodium and perfused through the heart with 4% PFA (Sigma‐Aldrich, St. Louis, MO, USA, 47 608). We used 30% sucrose‐formalin solution to fix the sample for one week. The tissue was paraffin‐embedded and sectioned by semi‐automatic low‐temperature slicer (KD‐2850, Jinhua, China) to a thickness of 4 mm. We then dewaxed the sections to water, stained the nuclei with haematoxylin and stained the cytoplasm with eosin. The samples were sealed with neutral gum. We examined the morphology of vertebral nerve cells in rat CA1 areas under the microscope (Nikon, Tokyo, Japan). The percentage of damaged nerve cells in hippocampus CA1 region was used as an evaluation indicator.

### 2.9 Neuropathological evaluation

The evaluation criteria for neuropathology were as follows: neuronal density (ND) was evaluated by counting the number of neuronals per 1 mm linear length in the hippocampal CA1 area according to of the methods of Xin et al^25^: The average number of nerve cells in three regions of hippocampal CA1 was accounted to evaluate the ND value. Histological grade (HG) was divided into four classes: no nerve cell death, grade 0; scattered single nerve cell death, grade I; mass nerve cell death, grade II; and almost complete nerve cell death, grade III.

#### 2.9.1 Immunofluorescence staining

To further identify the MEF2D, GFAP and caspase‐3 expression, we used immunofluorescence. Paraffin sections were dewaxed, and we repaired antigen and removed endogenous peroxidase. 0.2% Triton X‐100 and 5% bovine serum albumin (BSA) were used to block for 1 h, and sections were incubated at 4°C overnight with anti‐MEF2D (1:200, Abcam, Cambridge, UK, ab246884), anti‐GFAP (1:200, Abcam, Cambridge, UK, ab7260) and anti‐caspase‐3 (1:200, Abcam, Cambridge, UK, ab13847). Then, the sections were incubated with secondary antibody (FITC; 1:100, Abcam, Cambridge, UK, ab150077) for 1 h and stained with 4',6‐diamidino‐2‐phenylindole (DAPI) or propidium iodide (PI) for 6 min in the dark. Finally, the images were captured immediately by the confocal laser scanning microscope (Zeiss, Jena, Germany). Mean fluorescence intensity was used to evaluate the level of each protein expression.

#### 2.9.2 Neuronal apoptosis assay

The TdT‐mediated dUTP Nick‐End Labelling (TUNEL) technique was used to evaluate neuronal apoptosis. The In Situ Cell Death Detection Kit (Roche, Switzerland, Germany, 11 684 795 960) was used according to the instruction. The nuclei were stained with DAPI, and fluorescent microscopy (Olympus, Tokyo, Japan) was used to evaluate neuronal apoptosis.

#### 2.9.3 Western blotting

The proteins were separated from the infarcted hemisphere hippocampal tissue and then separated by 10% sodium dodecyl sulphate polyacrylamide gel electrophoresis (SDS‐PAGE) and then transferred to polyvinylidene fluoride (PVDF) membranes. 10% skimmed milk or BSA was used to block for 2 h, and we incubated the membranes at 4°C overnight with anti‐MEF2D (1:1000, Abcam, Cambridge, UK, ab246884), anti‐p‐ERK5 (1:1000, Abcam, Cambridge, UK, ab5686), anti‐ERK5 (1:1000, Abcam, Cambridge, UK, ab40809) and β‐actin (1:10,000, ZSGB‐BIO, Beijing, China, TA‐09). Next, we followed this with incubation with a secondary antibody labelled by horseradish peroxidase (1:10 000, ZSGB‐BIO, Beijing, China, ZB‐2301) for 2 h. Finally, we used enhanced chemiluminescent reagent (Thermo Fisher, Waltham, MA, USA, D1306) to examine blots, and ImageJ (Rawak Software Inc, Stuttgart, Germany) to analyse blots quantitatively.

#### 2.9.4 Quantitative Real‐Time Polymerase Chain Reaction (qRT‐PCR)

The expression level of synGAP and arc mRNA was detected by qRT‐PCR. Briefly, total RNA was extracted by TRIzol (Solarbio Life Science, 15 596 026, Beijing, China). The total RNA was reverse‐transcribed to cDNA using a Reverse Transcription System (Bioer, BSB09M1, Hangzhou, China).

Quantitative real‐time PCR was performed via the LightCycler480 Software (Thermo Fisher Scientific, Waltham, MA, USA) with Power SYBR Green (Thermo Fisher Scientific, Waltham, MA, USA). The cycling conditions were as follows: 2min at 95°C followed by 40 cycles of 15s at 95°C and 30s at 72°C. The expression of each targeted gene was quantified through the threshold cycle (Ct) method. The primer sequences were as follows: synGAP:5’‐TCGTGTTCAAGGAGACAGGC‐3’, 5’‐CAGGTGCTGGTTGCTTTTCC‐3’;arc:5’‐TATGTGGACGCTGAGGAGGA‐3’,5’‐CGCAGAAAGCGCTTGAACTT‐3’; and GAPDH: 5’‐CAGGGCTGCCTTCTCTTGTG‐3’, 5’‐AACTTGCCGTGGGTAGAGTC‐3’.

#### 2.9.5 Statistical analysis

All of the data are shown as mean ± SD. We used GraphPad Prism 7 (GraphPad Software, La Jolla, CA, USA) for statistical analyses, and the Kolmogorov‐Smirnov test was used to the normality of the results. Normally distributed data were analysed by Student's t test and one‐way analysis of variance (ANOVA). Non‐normally distributed data were analysed by nonparametric Mann‐Whitney *U* test or Kruskal‐Wallis with Dunn test. Statistical significance was expressed as *P* < .05.

## 3 RESULTS

### 3.1 Cerebral I/R injury reduces MEF2D expression in hippocampal CA1 area

**FIGURE 1:**
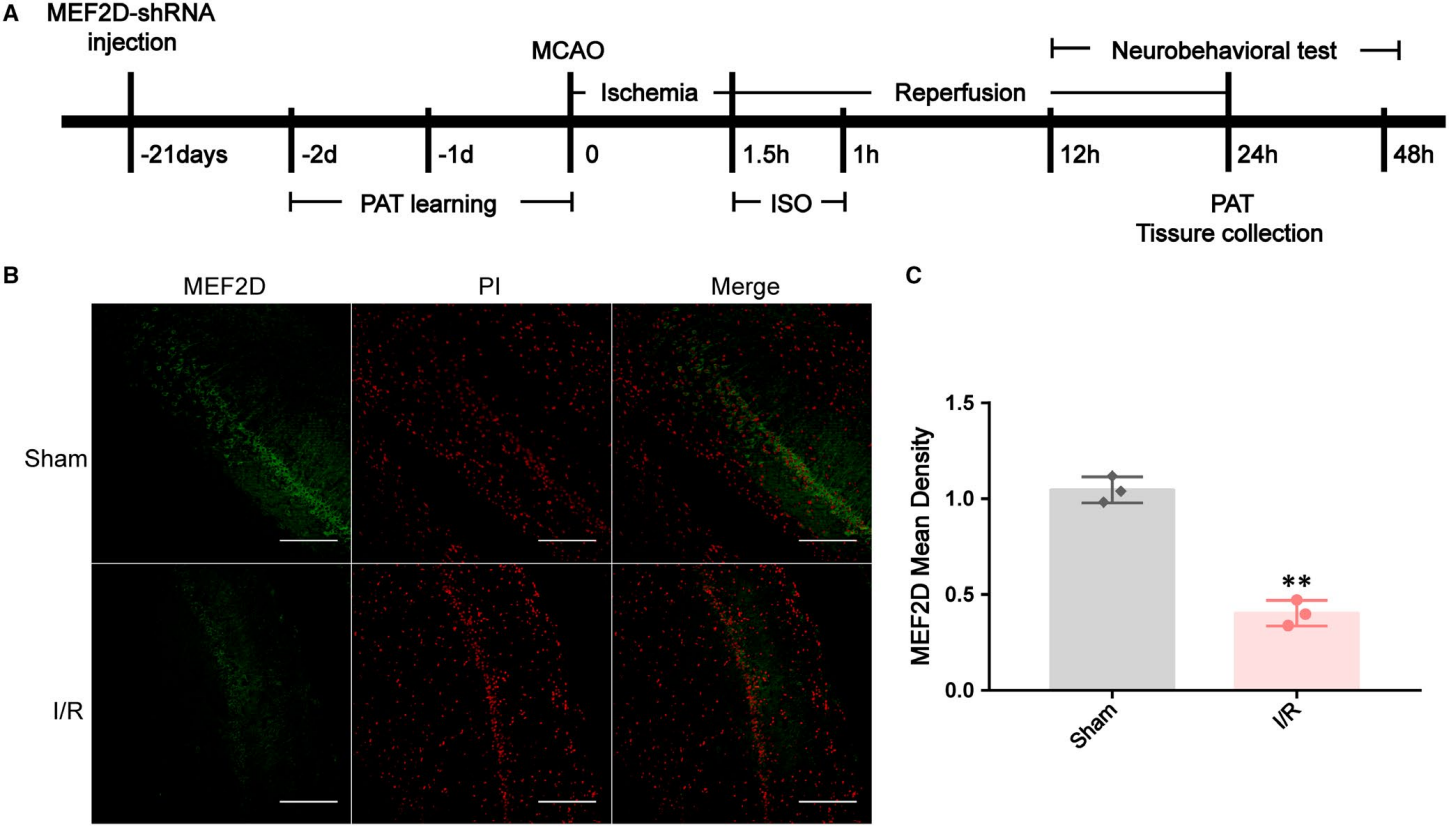
(A) Overall experimental process. MCAO, middle cerebral artery occlusion; PAT, passive avoidance test, ISO, isoflurane. (B, C) Cerebral I/R injury reduces MEF2D expression in hippocampal CA1 area. Data are shown as mean ± SD, n = 3. **P < .01. Original magnification: 100×. Scale bar was 50 μm

To test the reactivity of MEF2D to stroke, MCAO was performed for 90 min, followed by 24 h of reperfusion. Next, MEF2D expression following stroke was evaluated by co‐staining MEF2D with PI in the hippocampus. As shown in Figure 1A, immunofluorescence showed that the majority of MEF2D was expressed in hippocampal pyramidal neuronals. Compared with Sham group, brain I/R injury strongly reduced expression of MEF2D in hippocampus in I/R group (Sham: 1.046 ± 0.04, IR: 0.403 ± 0.04, *P* < .01, Figure 1B, C).

### 3.2 Knockdown of MEF2D leads to increased I/R‐induced cerebral damage and worsened post‐stroke outcome in rats

**FIGURE 2:**
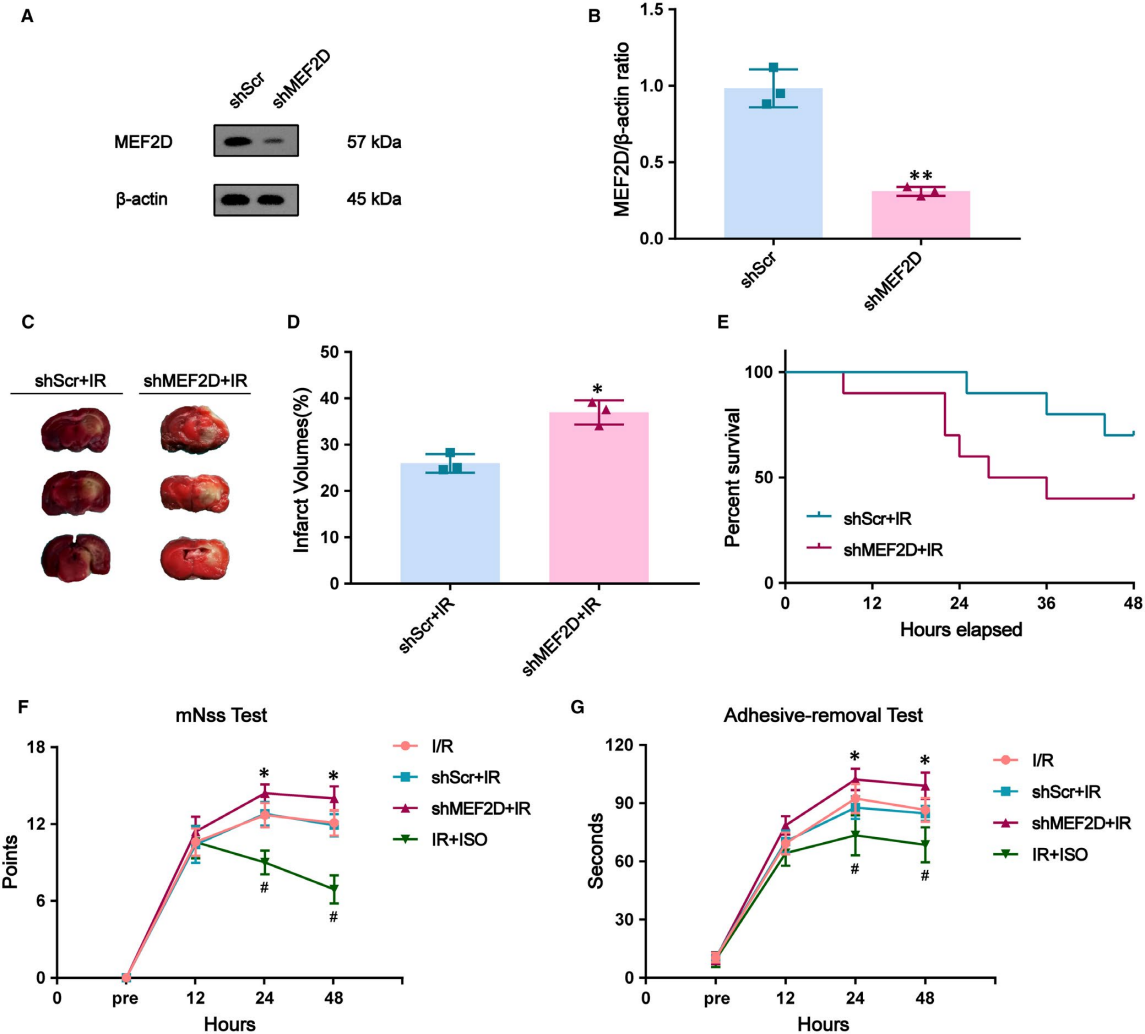
Impact of MEF2D deletion on brain survival and neurological defect after I/R injury in rats. (A, B) Western blot analysis shows down‐regulation in MEF2D expression in the hippocampus within 21 days after MEF2D‐shRNA injection, n = 3. (C) Brain ischaemia staining by TTC (red, non‐infarct part; white, infarct part). (D) Brain infarct volume shown as a percentage of the ischaemia volumes, n = 3. (E) After cerebral I/R injury, shMEF2D reduced survival at 48 h compared with the shScr + IR group, n = 10. (F, G) The mNSS and adhesive‐removal tests were executed 3 d before and 12, 24 and 48 h after brain I/R injury, n = 5‐10. Data are shown as mean ± SD, *P < .05, ^#^ *P* < .05

In vivo knockdown of MEF2D was performed by stereotactic injection of lentiviral vectors expressing MEF2D‐shRNA into the ischaemic hippocampus. First, we assessed MEF2D silencing efficiency by measuring MEF2D protein levels in the hippocampus. We found that shMEF2D rats exhibited reduced MEF2D protein expression compared with shScr rats at 21 days after shRNA injection (shMEF2D group: 0.31 ± 0.02, shsCR group: 0.98 ± 0.07, *P* < .01; Figure 2A, B). To evaluate the relationship of MEF2D in cerebral I/R injury, shMEF2D rats underwent MCAO. Importantly, shMEF2D + IR rats exhibited increased stroke size compared with shScr + IR rats at 24 hours after I/R as assessed by TTC staining (shMEF2D + IR group: 36.97 ± 1.51, shScr + IR group: 25.97 ± 1.17, *P* < .05; Figure 2C, D). Moreover, the survival rate 48 h after MCAO was reduced in shMEF2D + IR group as compared with the shScr + IR group (40% in the shMEF2D + IR group, 70% in the shScr + IR group, *P* < .01; Figure 2E). Neurological functions were quantified by mNSS and adhesive‐removal test before and 12, 24 and 48 h after I/R. We observed no differences in neurological functions between the groups at baseline. At 24 and 48 h after brain I/R injury, the neurological functions of the shMEF2D + IR group were lower compared with the shScr + IR group (*P* < .05; Figure 2F, G). There were no differences between the I/R group and the shScr + IR group.

### 3.3 Knockdown of MEF2D leads to worsen cognitive impairment after brain I/R injury

The passive avoidance test (PAT) is a simple tool that assesses the cognition function of rats. It can also effectively eliminate the influence of motor dysfunction on learning and memory observation indexes. As shown in figure A and B, after MCAO at 24 h, the I/R group displayed a reduced latency to enter into the darkroom compared with the Sham group (51.0 ± 2.24 s in the I/R group, 176.60 ± 4.13 s in Sham group, *P* < .01) and this was exacerbated by knockdown of MEF2D in shMEF2D + IR group compared with the shScr + IR group (27.60 ± 2.26 s in the shMEF2D + IR group, 52.80 ± 2.25 s in the shScr + IR group, *P* < .01). Furthermore, the I/R group made more error trials in entering the darkroom compared with the sham group (2.10 ± 0.18 in I/R group, 0.21 ± 0.13 in Sham group, *P* < .01), and this was significantly increased by knockdown of MEF2D in shMEF2D + IR group (2.80 ± 0.29 in the shMEF2D + IR group, 1.80 ± 0.25 in the shScr + IR group, *P* < .05). No differences between the I/R group and shScr + IR group. The results indicate that passive avoidance learning test in rats could be damaged by I/R injury. These effects, nevertheless, were further weakened by knockdown of MEF2D.

### 3.4 Knockdown of MEF2D increases neuronal apoptosis in hippocampal CA1 region

**FIGURE 3:**
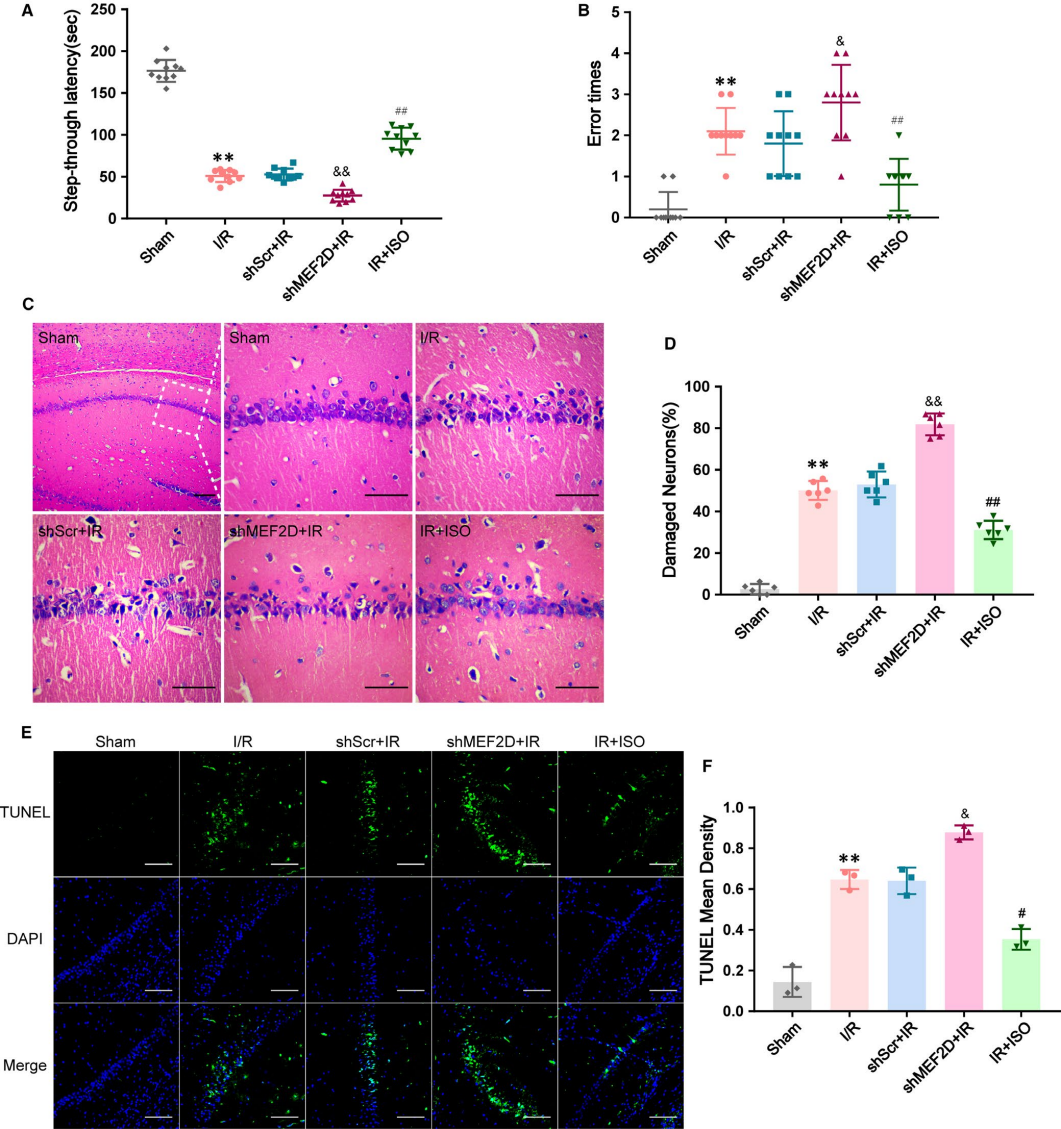
(A, B) Effects of MEF2D on cognition function of rats in PAT, n = 6‐10. (C) Effects of MEF2D on the morphological changes of CA1 area by HE staining, n = 6. (D) Percentage of damaged neuronals in each group. (E, F) Effects of MEF2D on apoptosis after brain I/R injury by TUNEL staining, n = 3. Data are presented as mean ± SD. **P < .01, compared to Sham group; ^&&^ *P* < .01, ^&^ *P* < .05, compared to shScr + IR group; ^##^ *P* < .01, ^#^ *P* < .05, compared to I/R group. Original magnification: 200×. Scale bar is 100 μm

To further elucidate the effect of MEF2D on apoptosis after I/R injury, TUNEL staining was used to assess the severity of apoptosis in hippocampal CA1 area after I/R. After I/R, neuronal apoptosis rate in CA1 region increased significantly when compared to Sham group (0.65 ± 0.03 in I/R group; 0.14 ± 0.04 in Sham group, *P* < .01). In comparing knockdown of MEF2D compared with the shScr + IR group, we observed that the neuronal apoptosis rate of shMEF2D + IR group was greater (0.88 ± 0.02 in shMEF2D + IR group; 0.64 ± 0.04 in shScr + IR group, *P* < .05; Figure 3E, F).

### 3.5 Isoflurane post‐conditioning reduced cognitive impairment after cerebral I/R injury

In PAT, the IR + ISO group revealed a reduced latency to enter into the darkroom compared with the I/R group (95.60 ± 4.10 in IR + ISO group, 51.00 ± 2.24 in I/R group, *P* < .01; Figure 3A) and fewer error times to enter into the darkroom (0.80 ± 0.20 in IR + ISO group, 2.10 ± 0.18 in I/R group, *P* < .01; Figure 3B).

### 3.6 Isoflurane post‐conditioning reduced the per cent of damaged neuronals and neuronal apoptosis after I/R injury in hippocampus

In HE staining, the IR + ISO group revealed a reduced percentage of dead neuronals in CA1 region compared with I/R group (31.12% ±1.81% in IR + ISO group, 50.10% ± 1.87% in I/R group, *P* < .01). neuronal apoptosis rate also showed a significant decrease compared with I/R group (0.35 ± 0.03 in IR + ISO group, 0.65 ± 0.03 in I/R group, *P* < .05; Figure 3C, D).

### 3.7 Isoflurane post‐conditioning increased the expression of protein MEF2D

**FIGURE 4:**
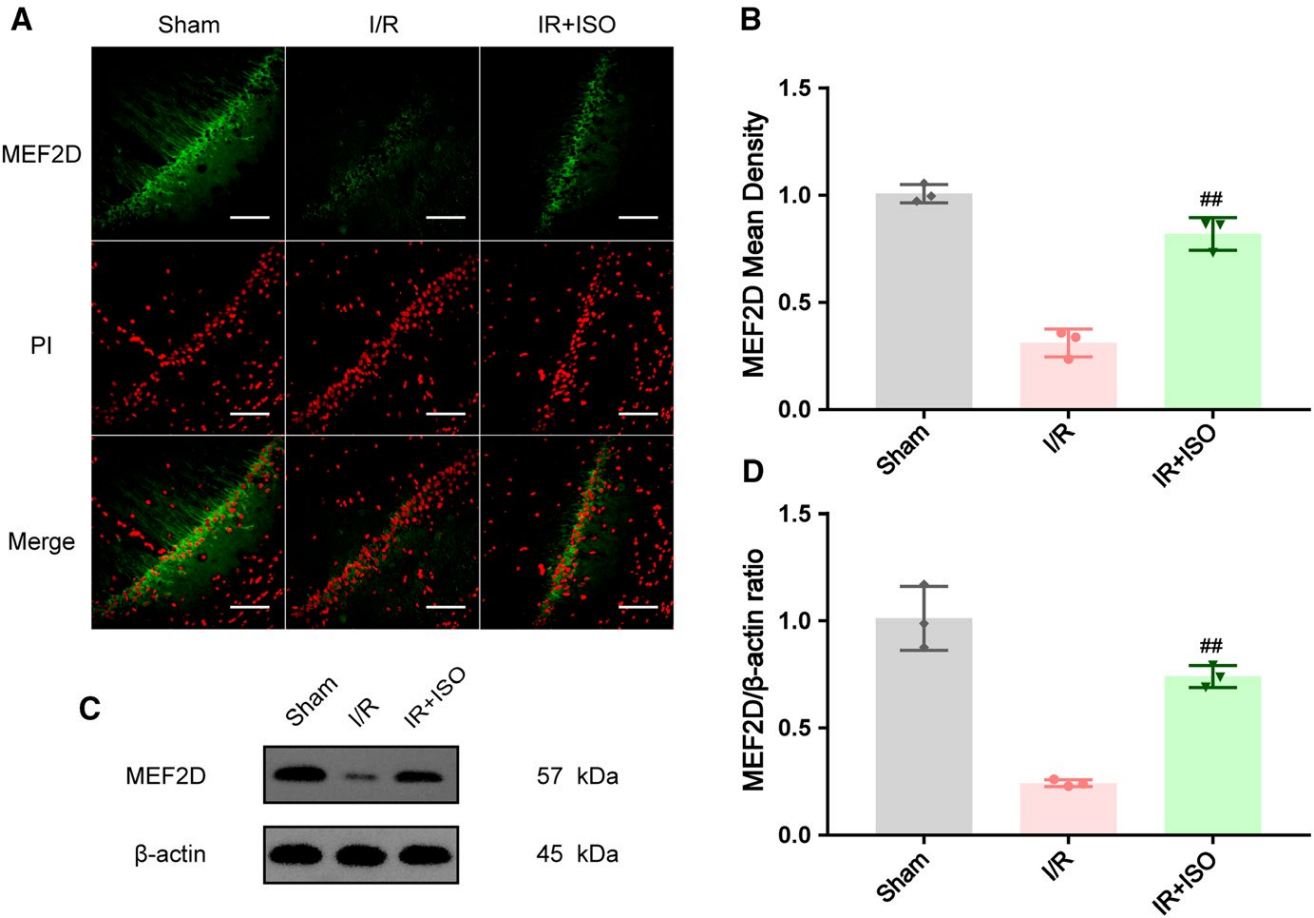
Expression of protein MEF2D shows significant increases through isoflurane post‐conditioning, n = 3. (A, B) Effects of ISO post‐treatment on MEF2D expression in CA1 region after I/R injury by immunofluorescence. (C, D) Effects of ISO post‐treatment on MEF2D expression after brain I/R injury by western blotting, n = 3. Data are shown as mean ± SD. ^##^ *P* < .01, compared to I/R group. Original magnification: 200×. Scale bar is 100 μm

The expression of protein MEF2D increased significantly through ISO post‐conditioning. We detected expression of MEF2D by using Western blotting and immunofluorescence. As shown in Figure 4, the MEF2D mean fluorescence density was increased compared with I/R group (0.82 ± 0.04 in IR + ISO group; 0.31 ± 0.04 in I/R group, *P* < .01). In Western blot work, the expression of protein MEF2D also increased in IR + ISO group compared with I/R group (0.74 ± 0.03 in IR + ISO group; 0.24 ± 0.01 in I/R group, *P* < .01).

### 3.8 Isoflurane post‐conditioning increases the transcriptional activity of MEF2D by activating ERK5

**FIGURE 5:**
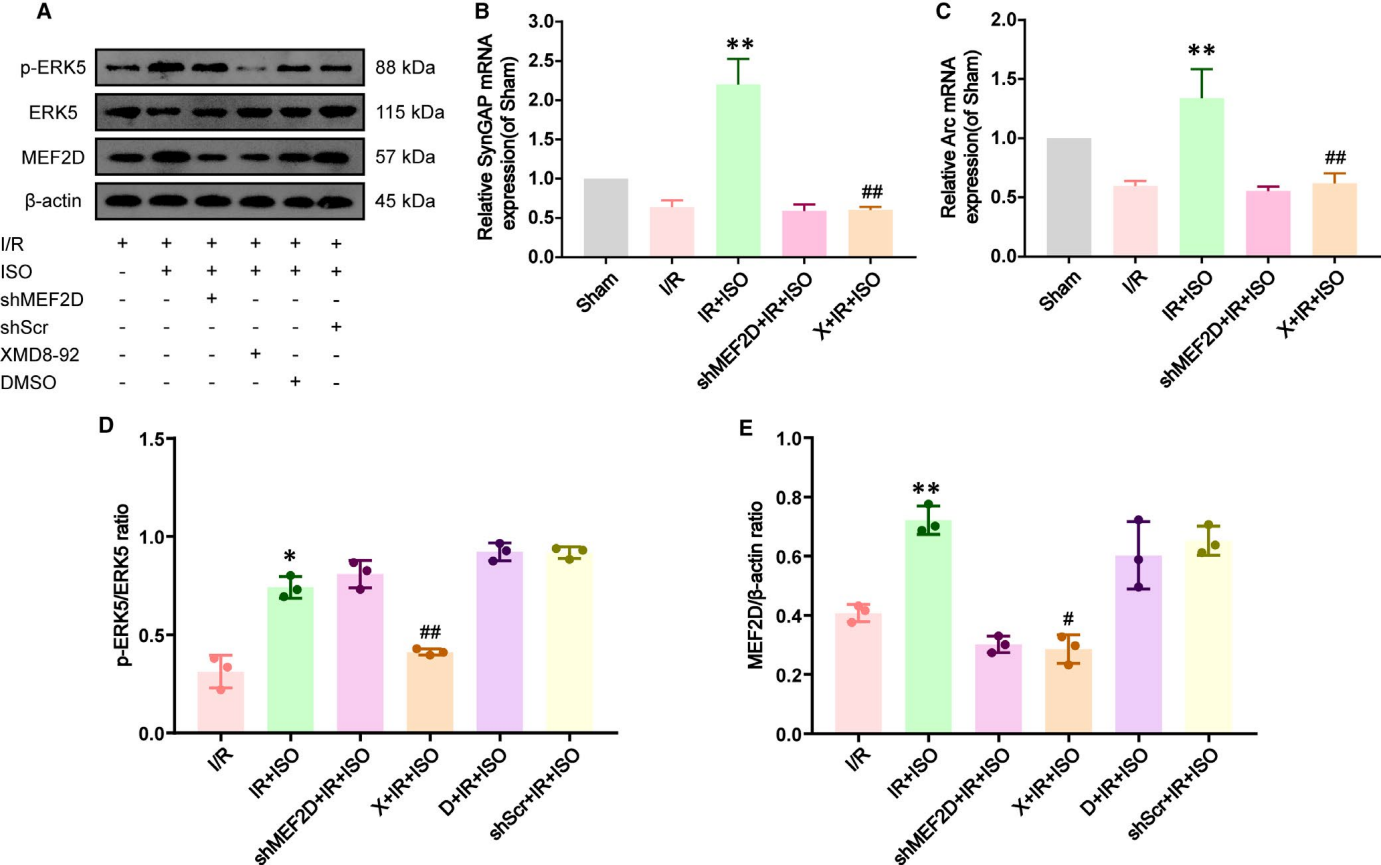
ISO post‐conditioning effect on ERK5, MEF2D and its target genes in hippocampus after brain I/R injury, n = 3. (A) Western blot staining of p‐ERK5, ERK5 and MEF2D. (B) Expression of synGAP mRNA, n* = *3. (C) Expression of arc mRNA, n* = *3. (D) The p‐ERK5/ERK5 expression level. (E) MEF2D expression level. Data are shown as mean ± SD. ^##^ *P* < .01, ^#^ *P* < .05, compared to IR + ISO group; **P < .01, *P < .05, compared to I/R group

We investigated the changes in ERK5, MEF2D and its downstream target genes in ISO post‐conditioning through Western blot and qRT‐PCR. To evaluate the extent of neuronal defects caused by loss of ERK5‐MEF2D signalling in the context of no I/R injury, we found that the shMEF2D and XMD8‐92 treatment of sham‐treated had no effect on apoptosis of SD rats (Figure S1). As shown in Figure 5, we observed that ISO post‐conditioning increased p‐ERK5 and promoted the activation of ERK5, compared with the I/R group (0.74 ± 0.03 in IR + ISO group; 0.31 ± 0.05 in I/R group, *P* < .05), while MEF2D expression was significantly up‐regulated (0.72 ± 0.03 in IR + ISO group; 0.41 ± 0.02 in I/R group, *P* < .05), and the transcription of two target genes related to synaptic plasticity regulated by MEF2D was enhanced (synGAP: 2.20 ± 0.19 in IR + ISO group; 0.63 ± 0.05 in I/R group, *P* < .05; arc: 1.34 ± 0.14 in IR + ISO group; 0.60 ± 0.02 in I/R group, *P* < .05). However, this effect can be significantly inhibited by XMD8‐92, as XMD8‐92 is a specific and highly selective inhibitor of ERK5. After XMD8‐92 was injected in the lateral ventricle of the model, ERK5 activation was more inhibited after brain I/R injury compared with ISO group (0.41 ± 0.01 in X + IR+ISO group; 0.74 ± 0.03 in IR + ISO group; *P* < .01). Moreover, the activation effect of isoflurane on MEF2D disappeared (0.29 ± 0.03 in X + IR+ISO group; 0.72 ± 0.03 in IR + ISO group, *P* < .01). The transcriptional activity of MEF2D decreased (synGAP: 0.60 ± 0.12 in X + IR+ISO group; 2.20 ± 0.18 in IR + ISO group, *P* < .05; arc: 0.62 ± 0.05 in X + IR+ISO group; 1.34 ± 0.14 in IR + ISO group, *P* < .05). After down‐regulating the expression of MEF2D, ERK5 activation was not affected by isoflurane compared with the IR + ISO group (0.81 ± 0.04 in shMEF2D + IR+ISO group; 0.74 ± 0.03 in IR + ISO group, *P* = .26).

### 3.9 Isoflurane post‐treatment increased the number of viable vertebral neuronals in hippocampus after I/R injury through the ERK5/MEF2D signalling pathway in rats

**FIGURE 6:**
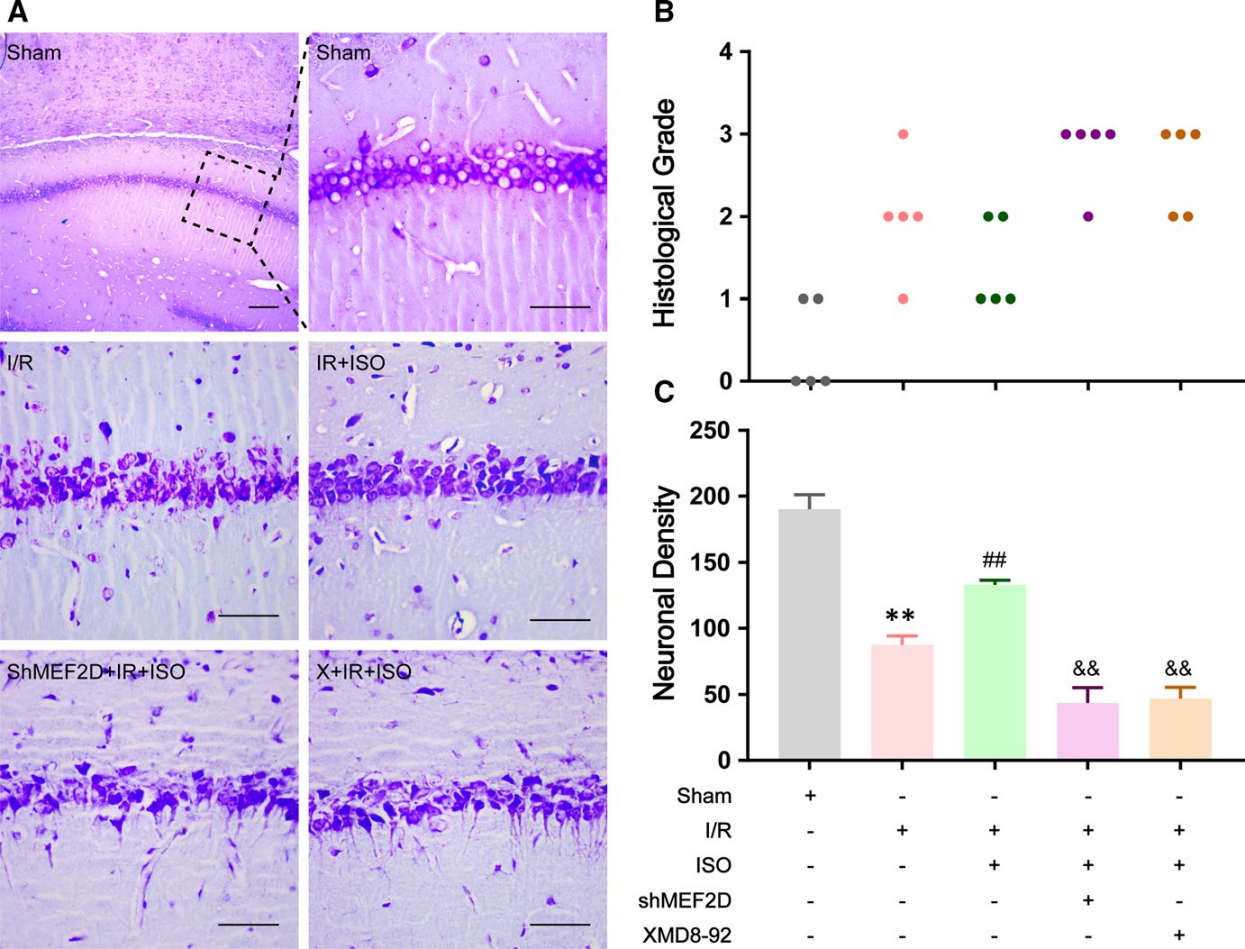
(A) Effects of the ERK5/MEF2D signalling pathway on neuronal survival in ISO post‐treatment after brain I/R injury by Nissl's staining, n = 5. (B, C) The results are a quantitative presentation of the evaluation with histological grade and neuronal density. Data are presented as mean ± SD. **P < .01, compared to Sham group, ^##^ *P* < .01, compared to I/R group, ^&&^ *P* < .01, compared to IR + ISO group. Scale bar is 100 μm

After I/R injury at 24 h, Nissl's staining method was used to evaluate the relationship between the morphologic changes in vertebral neuronals in hippocampus after ISO post‐conditioning and the ERK5/MEF2D signalling pathway. As shown in Figure 6, cells in CA1 are characterized by the hyperchromatic nucleus, with pyknosis and shaped into triangle and diamond. Compared to Sham group, the surviving nerve cell count in I/R group was decreased observably (87.67 ± 3.84 in I/R group; 190 ± 6.43 in Sham group, *P* < .01). After isoflurane post‐conditioning however, surviving neuronals increased significantly (133 ± 2.08 in IR + ISO group; 87.67 ± 3.84 in I/R group, *P* < .01). However, the influences of ISO post‐conditioning on surviving neuronals were attenuated by shMEF2D and XMD8‐92 (43.67 ± 6.69 in shMEF2D + IR+ISO group; 47 ± 4.93 in X + IR+ISO group; *P* < .01).

#### 3.9.1 Isoflurane post‐conditioning reduced the expression of astrocytes in hippocampal after I/R injury through the ERK5/MEF2D signalling pathway in rats

**FIGURE 7:**
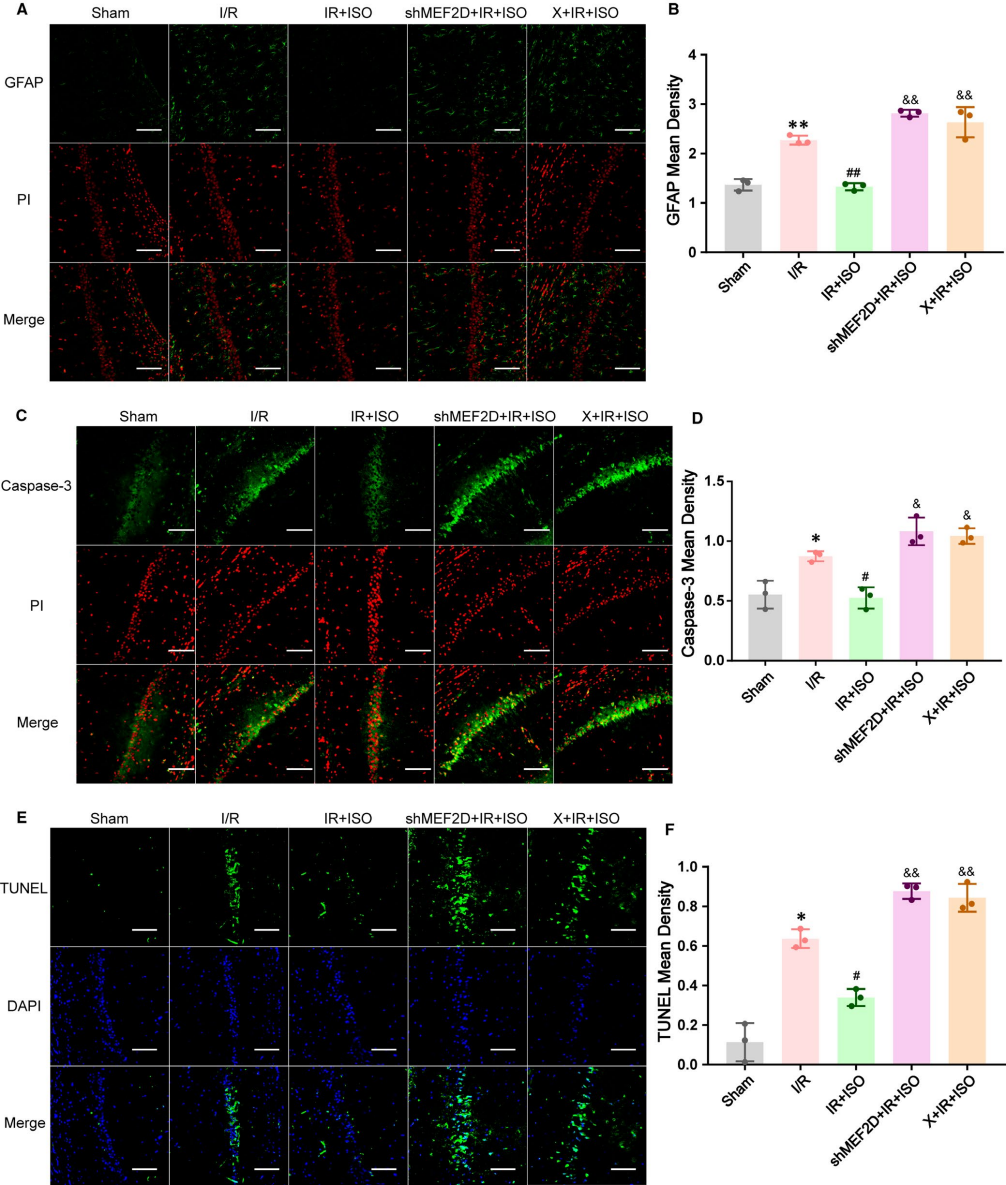
(A, B) Expression of GFAP in hippocampus after ISO post‐conditioning I/R injury. (C, D) Effects of ERK5/MEF2D signalling pathway on caspase‐3 expression in ISO post‐conditioning after brain I/R injury by immunofluorescence. (E, F) Effects of ERK5/MEF2D signalling pathway on apoptosis in ISO post‐conditioning after I/R injury by TUNEL staining, n = 3. Data are presented as mean ± SD. **P < .01, *P < .05, compared to Sham group, ^##^ *P* < .01, ^#^ *P* < .05, compared to I/R group, ^&&^ *P* < .01, ^&^ *P* < .05, compared to IR + ISO group. Original magnification: 200×. Scale bar is 100 μm

To elucidate the relationship between the ERK5/MEF2D signalling pathway and astrocyte activation in I/R injury treated with ISO post‐conditioning, we used GFAP to assess immunofluorescent staining for reactive astrocyte marker. As shown in Figure 7A and B, green fluorescence indicates the GFAP, and red fluorescence indicates the cell nucleus. 24 h after brain I/R injury, GFAP‐positive cells increased significantly in the hippocampus compared with Sham group (2.27 ± 0.05 in I/R group; 1.37 ± 0.07 in Sham group, *P* < .01). When treated with isoflurane post‐conditioning, GFAP‐positive cells decreased significantly (1.33 ± 0.04 in IR + ISO group; 2.27 ± 0.05 in I/R group, *P* < .01). However, both MEF2D and ERK5 silencing significantly blocked the action of ISO post‐conditioning (2.82 ± 0.04 in shMEF2D + IR+ISO group; 2.63 ± 0.18 in X + IR+ISO group; *P* < .01), compared with the IR + ISO group.

#### 3.9.2 Anti‐apoptotic effect of ERK5/MEF2D signalling pathway on post‐conditioning of isoflurane after brain I/R injury in rats

To determine whether ERK5/MEF2D signalling pathway inhibition attenuates the protective influence on ISO post‐conditioning after brain I/R injury, we used immunofluorescence double‐labelling to detect the expression of caspase‐3 and TUNEL‐positive signals in hippocampal CA1 to evaluate the severity of apoptosis. As shown in figure C and E, compared to Sham group, caspase‐3 and TUNEL expression increased markedly in I/R group (caspase‐3:0.87 ± 0.02 in I/R group; 0.55 ± 0.07 in Sham group, *P* < .05; TUNEL: 0.64 ± 0.03 in I/R group; 0.11 ± 0.06 in Sham group, *P* < .05; Figure 7D, F). After ISO post‐conditioning, caspase‐3 and TUNEL expression decreased in IR + ISO group (both *P* < .05). However, the anti‐apoptotic effects of ISO post‐conditioning were attenuated by MEF2D‐shRNA (caspase‐3:1.08 ± 0.07 in shMEF2D + IR+ISO group, *P* < .05; TUNEL: 0.88 ± 0.02 in shMEF2D + IR+ISO group, *P* < .01) and XMD8‐92 (caspase‐3:1.04 ± 0.04 in X + IR+ISO group, *P* < .05; TUNEL: 0.84 ± 0.04 in X + IR+ISO group, *P* < .01), compared to IR + ISO group.

## 4 DISCUSSION

Ischaemic cerebral stroke is the first cause of death worldwide, and its prevalence may increase due to the changing epidemiology of diabetes, coronary heart disease (CHD) and hypertension.^26^ In order to deepen our understanding of molecular networks in ischaemic stroke and identify new therapeutic targets, we have previously investigated the role of different drug interventions in activating the corresponding signalling pathways in the occurrence of stroke.^27^, ^28^, ^29^ Recently, MEF2D has been reported as a target in promoting cell proliferation in humans^30^ and appears to play a crucial role in systemic neuroprotection^31^ and normal sleep and circadian behaviour in mice.^32^ In view of the key role of MEF2D in neuroprotection and the correlation between nerve injury and prognosis in patients with ischaemic stroke, we studied the specific function of MEF2D in ischaemic cerebral stroke in rat model of I/R injury.

We have demonstrated that MEF2D improves prognosis after I/R injury and may be an interesting new therapeutic target. This conclusion is supported by the results showing that: (i) the expression of MEF2D prominently decreased in the hippocampus of rats after brain I/R injury; (ii) MEF2D silencing increased the infarct volume of ischaemic stroke rats and decreased the neurological impairment and short‐term survival rate of rats after stroke by exacerbating apoptosis; and (iii) MEF2D silencing increased cognitive dysfunction in ischaemic stroke rats.

There is abundant evidence that promoting the expression of MEF2D is considered to have a neuroprotective effect in vivo and *in vitro*,^33^ and the down‐regulation of MEF2D shows negative effects, such as PC12 cell death,^34^ subarachnoid haemorrhage^35^ and Parkinson's disease.^36^ Given the key role of hippocampal tissue damage in the prognosis of stroke, we focused in this study on MEF2D and found that its level significantly reduced in hippocampal tissue in the CA1 area of the brain exposed to I/R. Furthermore, we found that the inhibition of MEF2D aggravated the cognitive dysfunction of rats after 24 h of ischaemic reperfusion injury. These findings suggest that MEF2D may be involved in I/R‐mediated brain damage and add new information to the hippocampal hypoxia response. Although this is in keeping with previous studies on other animal models,^37^, ^38^ the exact mechanisms that mediate MEF2D reduction in ischaemic and hypoxic conditions are not fully understood. One explanation is that this may be related to the MEF2s’ family, which contains crucial regulators of cellular remodelling.

When blood recanalization occurs after cerebral ischaemia, the reperfusion brain injury caused by intracellular Ca2 + overload,^39^ damage of free radicals and inflammatory cytokines,^40^ neurotoxic effects of excitatory amino acids,^41^ disorder of water electrolyte and other factors.^42^ These factors interact and eventually lead to severe oedema, necrosis and apoptosis of vertebral neuronal cells. In our previous work, we demonstrated that the concentration of 1.5% isoflurane post‐processing immediately after reperfusion significantly improved brain damage in rats after I/R injury.^21^ In this study, isoflurane post‐conditioning was also observed to improve neurological deficits and outcomes after I/R injury using neurodeficit scores, dark avoidance tests, and HE and TUNEL staining. However, the exact mechanism of isoflurane post‐conditioning related to activation of a variety of signalling pathways is unclear and merits further study.

Apoptosis is a process associated with cytokine release,^43^ and although MEF2D is an important regulator of nerve cell survival, the relationship between MEF2D and apoptosis is not well studied. It has been reported that MEF2D is a specific substrate of ERK5,^19^ and the synergistic activation of ERK5 and MEF2D plays a crucial role in the survival of myocardial cells.^44^ In our experiments, we found that ISO post‐conditioning activated ERK5 by inducing phosphorylation, phosphorylation of ERK5 promoted the transcriptional activity of MEF2D, and the use of XMD8‐92 or MEF2D‐shRNA eliminated the neuroprotective effect of ISO.

There is growing evidence that astrocytes play a crucial role in neuroinflammation caused by stroke, Alzheimer's disease, and postoperative cognitive dysfunction.^45^ Studies have shown that in the acute ischaemic phase, astrocytes are immediately activated, enabling the synthesis and release of chemokines and cytokines, triggering an inflammatory cascade that may exacerbate secondary brain injury.^46^ Reactive astrocytes, however, limit inflammatory cascades and secondary brain damage by forming glial scarring around infarction after cerebral ischaemia.^47^ Here, we found that ISO post‐conditioning inhibited the activation of astrocytes through the ERK5/MEF2D signalling pathway. Current studies on new glial cells have advantages and disadvantages in different stages of the disease. Regarding the dual role of reactive astrocytes in neurotoxicity and neuroprotection, we should cautiously and optimistically view glial cell therapy and further study the role of astrocytes in neurological diseases.

There are some limitations to the studies presented here. First, ISO is not a specific agonist of ERK5. As mentioned above, ISO can play a neuroprotective role through different processes, while the anti‐apoptotic effect of ISO may not be related to the direct activation of ERK5. However, the ERK5 /MEF2D pathway may contribute to the anti‐apoptotic effects of ISO partially. Second, MEF2D mainly plays a role in the nucleus. Due to the constraints of experimental equipment, we were only able to detect the total level of MEF2D, but not the nuclear level of MEF2D after ISO treatment. Finally, cognitive tests and neurodeficit scores were performed within two days, and long‐term observational studies were not possible. Follow‐up work in our laboratory will address these outstanding issues.

In conclusion, our experiments explored the role of MEF2D in the neuroprotection of brain I/R injury in rats, finding that the neuroprotective effects of ISO were related to the activation of ERK5/MEF2D signalling pathway to promote the transcriptional activity of MEF2D.

## CONFLICTS OF INTEREST

The authors declare that they have no conflict of interest.

## AUTHOR CONTRIBUTION

**Qingtong Zhang:** Conceptualization (lead); Data curation (equal); Formal analysis (lead); Methodology (equal); Writing‐original draft (lead); Writing‐review & editing (equal). **Jiangwen Yin:** Conceptualization (supporting); Data curation (equal); Formal analysis (equal); Methodology (equal). **Feng Xu:** Conceptualization (supporting); Data curation (equal); Methodology (lead). **Jingwen Zhai:** Data curation (equal). **Jieting Yin:** Data curation (equal); Methodology (equal). **Mingyue Ge:** Data curation (equal); Methodology (equal). **Wenyi Zhou:** Data curation (equal). **Nian Li:** Data curation (equal). **Xinlei Qin:** Data curation (equal). **Yan Li:** Project administration (equal). **Sheng Wang:** Conceptualization (supporting); Project administration (supporting); Writing‐review & editing (lead).

## ETHICS APPROVAL

The processing procedures of all animals were according to the National Institutes of Health Guide for the Care and Use of Laboratory Animals (NIH publications number 80‐23, revised in 1996). Study design and experimental protocols were approved by the Animal Care and Use Committee of the First Affiliated Hospital of the Medical College, Shihezi University.

## Supporting information

Fig S1

## Data Availability

We declared that materials described in the manuscript, including all relevant raw data, will be freely available to any scientist wishing to use them for non‐commercial purposes, without breaching participant confidentiality.

## References

[1] Amantea D , Bagetta G . Excitatory and inhibitory amino acid neurotransmitters in stroke: from neurotoxicity to ischemic tolerance. Curr Opin Pharmacol. 2017;35:111‐119. 28826602 10.1016/j.coph.2017.07.014

[2] Angelopoulou E , Pyrgelis E‐S , Piperi C . Neuroprotective potential of chrysin in Parkinson's disease: Molecular mechanisms and clinical implications. Neurochem Int. 2020;132:104612. 31785348 10.1016/j.neuint.2019.104612

[3] Bhattacharya P , Pandey AK , Paul S , Patnaik R . Melatonin renders neuroprotection by protein kinase C mediated aquaporin‐4 inhibition in animal model of focal cerebral ischemia. Life Sci. 2014;100:97‐109. 24530291 10.1016/j.lfs.2014.01.085

[4] Bickler PE , Zhan X , Fahlman CS . Isoflurane preconditions hippocampal neuronals against oxygen‐glucose deprivation: role of intracellular Ca2+ and mitogen‐activated protein kinase signaling. Anesthesiology. 2005;103:532‐539. 16129978 10.1097/00000542-200509000-00016

[5] Chen Z‐W , Liu A , Liu Q , et al. MEF2D mediates the neuroprotective effect of methylene blue against glutamate‐induced oxidative damage in HT22 hippocampal cells. Mol Neurobiol. 2017;54:2209‐2222. 26941101 10.1007/s12035-016-9818-1

[6] Cheon SY , Kim SY , Kam EH ,, et al. Isoflurane preconditioning inhibits the effects of tissue‐type plasminogen activator on brain endothelial cell in an in vitro model of ischemic stroke. Int J Med Sci. 2017;14:425‐433. 28539818 10.7150/ijms.18037 PMC5441034

[7] Cui H‐X , Chen J‐H , Li J‐W , Cheng F‐R , Yuan K . Protection of anthocyanin from myrica rubra against cerebral ischemia‐reperfusion injury via modulation of the TLR4/NF‐κB and NLRP3 Pathways. Molecules. 2018;23:1788. 10.3390/molecules23071788 PMC6099489 30036952

[8] Cui X , Li L , Hu Y‐Y , Ren S , Zhang M , Li W‐B . Sulbactam plays neuronal protective effect against brain ischemia via upregulating GLT1 in Rats. Mol Neurobiol. 2015;51:1322‐1333. 25064054 10.1007/s12035-014-8809-3

[9] DeVries AC , Nelson RJ , Traystman RJ , Hurn PD . Cognitive and behavioral assessment in experimental stroke research: will it prove useful? Neurosci Biobehav Rev. 2001;25:325‐342. 11445138 10.1016/s0149-7634(01)00017-3

[10] Diaz‐Cañestro C , Reiner MF , Bonetti NR , et al. AP‐1 (activated protein‐1) transcription factor JunD regulates ischemia/reperfusion brain damage via IL‐1β (Interleukin‐1β). Stroke. 2019;50:469‐477. 30626291 10.1161/STROKEAHA.118.023739

[11] Ekker MS , Verhoeven JI , Vaartjes I , Jolink WMT , Klijn CJM , de Leeuw F‐E . Association of stroke among adults aged 18 to 49 years with long‐term mortality. JAMA. 2019;321:2113‐2123. 31121602 10.1001/jama.2019.6560 PMC6547225

[12] Flippo KH , Gnanasekaran A , Perkins GA , et al. AKAP1 protects from cerebral ischemic stroke by inhibiting Drp1‐dependent mitochondrial fission. J Neurosci. 2018;38:8233‐8242. 30093535 10.1523/JNEUROSCI.0649-18.2018 PMC6146498

[13] Gao LI , She H , Li W , et al. Oxidation of survival factor MEF2D in neuronal death and Parkinson's disease. Antioxid Redox Signal. 2014;20:2936‐2948. 24219011 10.1089/ars.2013.5399 PMC4038998

[14] Guo B , Hu S , Zheng C ,, et al. Substantial protection against MPTP‐associated Parkinson's neurotoxicity in vitro and in vivo by anti‐cancer agent SU4312 via activation of MEF2D and inhibition of MAO‐B. Neuropharmacology. 2017;126:12‐24. 28807675 10.1016/j.neuropharm.2017.08.014

[15] Halder SK , Ueda H . Amlexanox inhibits cerebral ischemia‐induced delayed astrocytic high‐mobility group box 1 release and subsequent brain damage. J Pharmacol Exp Ther. 2018;365:27‐36. 29330155 10.1124/jpet.117.245340

[16] Han J , Zhang J‐Z , Zhong Z‐F , et al. Gualou Guizhi decoction promotes neurological functional recovery and neurogenesis following focal cerebral ischemia/reperfusion. Neural Regen Res. 2018;13:1408‐1416. 30106053 10.4103/1673-5374.235296 PMC6108212

[17] Han Y , Seyfried D , Meng Y , et al. Multipotent mesenchymal stromal cell‐derived exosomes improve functional recovery after experimental intracerebral hemorrhage in the rat. J Neurosurg. 2018;131:290‐300. 30028267 10.3171/2018.2.JNS171475

[18] He G , Xu W , Tong L , et al. Gadd45b prevents autophagy and apoptosis against rat cerebral neuronal oxygen‐glucose deprivation/reperfusion injury. Apoptosis. 2016;21:390‐403. 26882903 10.1007/s10495-016-1213-x

[19] Hu S , Mak S , Zuo X , Li H , Wang Y , Han Y . Neuroprotection against MPP(+)‐induced cytotoxicity through the activation of PI3‐K/Akt/GSK3β/MEF2D signaling pathway by rhynchophylline, the major tetracyclic oxindole alkaloid isolated from uncaria rhynchophylla. Front Pharmacol. 2018;9:768. 30072894 10.3389/fphar.2018.00768 PMC6060423

[20] Jin K , Xiang M . Transcription factor Ptf1a in development, diseases and reprogramming. Cell Mol Life Sci. 2019;76:921‐940. 30470852 10.1007/s00018-018-2972-z PMC11105224

[21] Kasler HG , Victoria J , Duramad O , Winoto A . ERK5 is a novel type of mitogen‐activated protein kinase containing a transcriptional activation domain. Mol Cell Biol. 2000;20:8382‐8389. 11046135 10.1128/mcb.20.22.8382-8389.2000 PMC102145

[22] Kim M‐K , Kim S‐C , Kang J‐I , et al. 6‐Hydroxydopamine‐induced PC12 cell death is mediated by MEF2D down‐regulation. Neurochem Res. 2011;36:223‐231. 21057871 10.1007/s11064-010-0309-x

[23] Kimura TE , Jin J , Zi M , et al. Targeted deletion of the extracellular signal‐regulated protein kinase 5 attenuates hypertrophic response and promotes pressure overload‐induced apoptosis in the heart. Circ Res. 2010;106:961‐970. 20075332 10.1161/CIRCRESAHA.109.209320 PMC3003662

[24] Komotar RJ , Kim GH , Sughrue ME , et al. Neurologic assessment of somatosensory dysfunction following an experimental rodent model of cerebral ischemia. Nat Protoc. 2007;2:2345‐2347. 17947976 10.1038/nprot.2007.359

[25] Lemoine S , Tritapepe L , Hanouz JL , Puddu PE . The mechanisms of cardio‐protective effects of desflurane and sevoflurane at the time of reperfusion: anaesthetic post‐conditioning potentially translatable to humans? Br J Anaesth. 2016;116:456‐475. 26794826 10.1093/bja/aev451

[26] Liberale L , Carbone F , Montecucco F , et al. Ischemic stroke across sexes: what is the status quo? Front Neuroendocrinol. 2018;50:3‐17. 29753797 10.1016/j.yfrne.2018.05.001

[27] Liberale L , Gaul DS , Akhmedov A , et al. Endothelial SIRT6 blunts stroke size and neurological deficit by preserving blood‐brain barrier integrity: a translational study. Eur Heart J. 2020;41(16):1575‐1587. 31603194 10.1093/eurheartj/ehz712

[28] Mohawk JA , Cox KH , Sato M , et al. neuronal Myocyte‐Specific Enhancer Factor 2D (MEF2D) is required for normal circadian and sleep behavior in mice. J Neurosci. 2019;39:7958‐7967. 31420455 10.1523/JNEUROSCI.0411-19.2019 PMC6774416

[29] Na J‐Y , Song K , Lee J‐W , Kim S , Kwon J . Pretreatment of 6‐shogaol attenuates oxidative stress and inflammation in middle cerebral artery occlusion‐induced mice. Eur J Pharmacol. 2016;788:241‐247. 27346834 10.1016/j.ejphar.2016.06.044

[30] Ortega E , Rengachari S , Ibrahim Z , et al. Transcription factor dimerization activates the p300 acetyltransferase. Nature. 2018;562:538‐544. 30323286 10.1038/s41586-018-0621-1 PMC6914384

[31] Pazyra‐Murphy MF , Hans A , Courchesne SL , et al. A retrograde neuronal survival response: target‐derived neurotrophins regulate MEF2D and bcl‐w. J Neurosci. 2009;29:6700‐6709. 19458239 10.1523/JNEUROSCI.0233-09.2009 PMC2709981

[32] Peng LI , Yang C , Yin J , et al. TGF‐β2 induces Gli1 in a Smad3‐dependent manner against cerebral ischemia/reperfusion injury after isoflurane post‐conditioning in rats. Front Neurosci. 2019;13:636. 31297044 10.3389/fnins.2019.00636 PMC6608402

[33] Pistritto G , Trisciuoglio D , Ceci C , Garufi A , D'Orazi G . Apoptosis as anticancer mechanism: function and dysfunction of its modulators and targeted therapeutic strategies. Aging. 2016;8:603‐619. 27019364 10.18632/aging.100934 PMC4925817

[34] Salma J , McDermott JC . Suppression of a MEF2‐KLF6 survival pathway by PKA signaling promotes apoptosis in embryonic hippocampal neuronals. J Neurosci. 2012;32:2790‐2803. 22357862 10.1523/JNEUROSCI.3609-11.2012 PMC6621893

[35] Sharma M , Hart RG , Connolly SJ , et al. Stroke outcomes in the COMPASS trial. Circulation. 2019;139:1134‐1145. 30667279 10.1161/CIRCULATIONAHA.118.035864

[36] Uzdensky AB . Apoptosis regulation in the penumbra after ischemic stroke: expression of pro‐ and antiapoptotic proteins. Apoptosis. 2019;24:687‐702. 31256300 10.1007/s10495-019-01556-6

[37] Wang B , Cai Z , Lu F , et al. Destabilization of survival factor MEF2D mRNA by neurotoxin in models of Parkinson's disease. J Neurochem. 2014;130:720‐728. 24848448 10.1111/jnc.12765

[38] Wang S , Yin J , Ge M , et al. Transforming growth‐beta 1 contributes to isoflurane postconditioning against cerebral ischemia‐reperfusion injury by regulating the c‐Jun N‐terminal kinase signaling pathway. Biomed Pharmacother. 2016;78:280‐290. 26898453 10.1016/j.biopha.2016.01.030

[39] Wang X , Tournier C . Regulation of cellular functions by the ERK5 signalling pathway. Cell Signal. 2006;18:753‐760. 16376520 10.1016/j.cellsig.2005.11.003

[40] Xiang J , Zhang NI , Sun H , et al. Disruption of SIRT7 increases the efficacy of checkpoint inhibitor via MEF2D regulation of programmed cell death 1 ligand 1 in hepatocellular carcinoma Cells. Gastroenterology. 2019;158(3):664‐678. 31678303 10.1053/j.gastro.2019.10.025

[41] Xu F , Ma R , Zhang G , et al. Estrogen and propofol combination therapy inhibits endoplasmic reticulum stress and remarkably attenuates cerebral ischemia‐reperfusion injury and OGD injury in hippocampus. Biomed Pharmacother. 2018;108:1596‐1606. 30372862 10.1016/j.biopha.2018.09.167

[42] Xu F , Zhang G , Yin J , et al. Fluoxetine mitigating late‐stage cognition and neurobehavior impairment induced by cerebral ischemia reperfusion injury through inhibiting ERS‐mediated neuronals apoptosis in the hippocampus. Behav Brain Res. 2019;370:111952. 31103751 10.1016/j.bbr.2019.111952

[43] Xu H , Li J , Wang Z , et al. Methylene blue attenuates neuroinflammation after subarachnoid hemorrhage in rats through the Akt/GSK‐3β/MEF2D signaling pathway. Brain Behav Immun. 2017;65:125‐139. 28457811 10.1016/j.bbi.2017.04.020

[44] Yang S , Gao LI , Lu F , et al. Transcription factor myocyte enhancer factor 2D regulates interleukin‐10 production in microglia to protect neuronal cells from inflammation‐induced death. J Neuroinflammation. 2015;12:33. 25890150 10.1186/s12974-015-0258-z PMC4339472

[45] Yu Q , Li L , Liang W‐M . Effect of sevoflurane preconditioning on astrocytic dynamics and neural network formation after cerebral ischemia and reperfusion in rats. Neural Regen Res. 2019;14:265‐271. 30531009 10.4103/1673-5374.244790 PMC6301166

[46] Yuan M , Ge M , Yin J , et al. Isoflurane post‐conditioning down‐regulates expression of aquaporin 4 in rats with cerebral ischemia/reperfusion injury and is possibly related to bone morphogenetic protein 4/Smad1/5/8 signaling pathway. Biomed Pharmacother. 2018;97:429‐438. 29091893 10.1016/j.biopha.2017.10.082

[47] Zhang G , Ge M , Han Z , et al. Wnt/β‐catenin signaling pathway contributes to isoflurane postconditioning against cerebral ischemia‐reperfusion injury and is possibly related to the transforming growth factorβ1/Smad3 signaling pathway. Biomed Pharmacother. 2019;110:420‐430. 30530044 10.1016/j.biopha.2018.11.143

