# Supplementary figures and images for: Isoflurane post‐conditioning contributes to anti‐apoptotic effect after cerebral ischaemia in rats through the ERK5/MEF2D signaling pathway
Source: J Cell Mol Med. 2021 Feb 23;25(8):3803–15. doi: 10.1111/jcmm.16282 (PMC8051747; doi:10.1111/jcmm.16282)

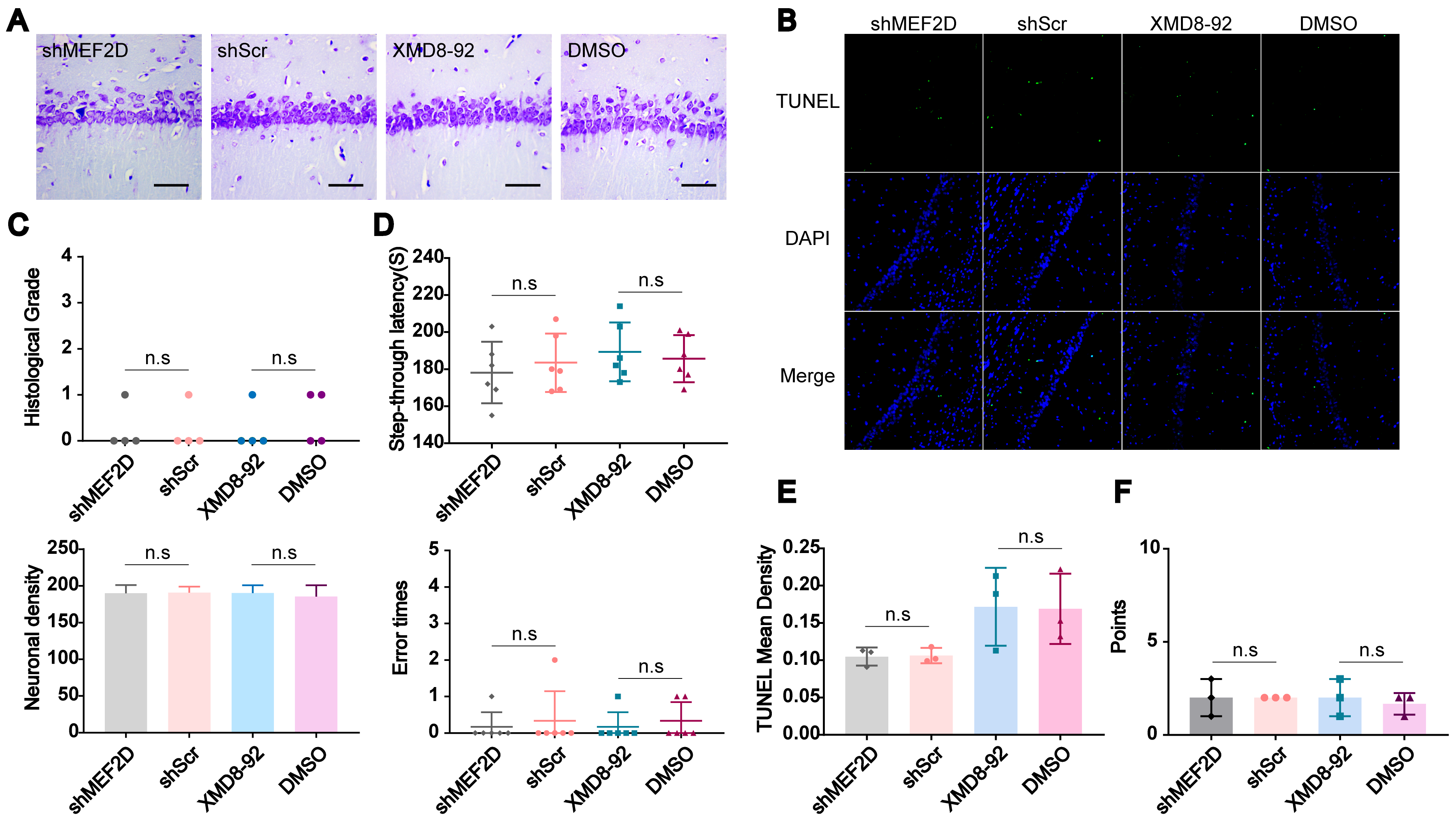

Supplement: Supplementary file 1 — Fig S1 [file JCMM-25-3803-s001.tif]
